# A qualitative study on older primary care patients’ perspectives on depression and its treatments - potential barriers to and opportunities for managing depression

**DOI:** 10.1186/s12875-017-0684-3

**Published:** 2018-01-03

**Authors:** Anne Stark, Hanna Kaduszkiewicz, Janine Stein, Wolfgang Maier, Kathrin Heser, Siegfried Weyerer, Jochen Werle, Birgitt Wiese, Silke Mamone, Hans-Helmut König, Jens-Oliver Bock, Steffi G. Riedel-Heller, Martin Scherer

**Affiliations:** 1 Institute of General Practice/Primary Care, University Medical Center Hamburg-Eppendorf, Martinistraße 52, 20246 Hamburg, Germany; 2 Institute of General Practice, Medical Faculty, Kiel University, Michaelisstr. 5, 24105 Kiel, Germany; 3 Institute of Social Medicine, Occupational Health and Public Health, Medical Faculty, University of Leipzig, Ph.-Rosenthal-Str. 55, 04103 Leipzig, Germany; 4 Department of Psychiatry and Psychotherapy, University of Bonn, Sigmund-Freud-Straße 25, 53105 Bonn, Germany; 5 Central Institute of Mental Health, Medical Faculty Mannheim/Heidelberg University, J 5, 68159 Mannheim, Germany; 6 Institute for General Practice, Working Group Medical Statistics and IT-Infrastructure, Hannover Medical School, Carl-Neuberg-Str. 1, 30625 Hannover, Germany; 7 Department of Health Economics and Health Services Research, Hamburg Center for Health Economics, University Medical Center Hamburg-Eppendorf, Martinistraße 52, 20246 Hamburg, Germany

**Keywords:** Depression in late life, Therapy, Older adults, Primary care, Patient perspective, Qualitative research

## Abstract

**Background:**

Depression is one of the most common mental disorders in old age and is associated with various negative health consequences for the affected individual. Studies suggest that patients’ views on depression have an impact on help-seeking behaviour and treatment. It is thus important to investigate the patient’s perspective in order to ascertain optimum management of depression in late life. However, studies on depression and its treatment exploring the perspectives of primary care patients 75 years or older, are rare.

**Methods:**

Qualitative data was collected in semi-structured interviews with 12 primary care patients 75 years of age or older with symptoms of depression. Data was analysed using qualitative content analysis.

**Results:**

The study’s results show the multifaceted views on and treatment of depression in primary care patients 75 years of age or older. Some patients seemed well informed about depression and believed in the efficacy of different treatments, such as medications or psychotherapy. However, some individuals had misconceptions about depression and its treatments. Patients mentioned that they would rather avoid talking about depression within their social network, in part of fear of negative reactions. Furthermore, participants believed that other people had little understanding for people with depression. Patients had different views on the relevance of the general practitioner’s (GP) role in treating depression; some patients believed that the GP had little importance in the treatment of depression.

**Conclusions:**

This study identified positive views of primary care patients 75 years of age or older towards depression as well as views that might hinder optimal treatments. Exemplary implications for an improved management of depression are: educating older adults about depression via age-specific information and having professionals encourage patients in believing that depression is a recognised disorder.

**Electronic supplementary material:**

The online version of this article (10.1186/s12875-017-0684-3) contains supplementary material, which is available to authorized users.

## Background

Depression is among the most common mental disorders in older adults [1, 2]. A pooled prevalence of depression of 17.1% [3] is described in the group of people 75 years of age or older. Depression in old age is associated with various negative health consequences for the affected individual, e.g., poorer health outcomes of medical conditions, functional and cognitive impairment or increased, non-suicidal mortality [4–7]. Despite effective treatments being available [8, 9], late-life depression is often stated to be under-recognised and undertreated. A recent analysis of German health insurance data indicates that the proportion of patients suffering from depression and who are not treated according to guidelines, increases with age. Moreover, with increasing age, only few patients receive psychotherapy [10]. Gum et al. found in a study with 1602 primary care patients that older adults preferred psychotherapy to antidepressants [11]. However, in contrast to younger adults, older adults seek any kind of professional help significantly less often for mental health problems [12]. Research identified different barriers faced by older adults seeking mental health treatment, e.g., the opinion that depression was normal in old age, transportation problems, insurance and payment concerns, lack of knowledge of how to access help or fear of stigmatisation [13–17].

Against this background, the improvement of care is an important objective, particularly in view of an increasing proportion of older adults in Germany and worldwide [18, 19]. In Germany, the number of individuals of the age of 80+ will almost triple and reach 11.6 million by 2050 [19] which means the number of individuals suffering from late-life depression might also triple. Different studies suggest that patients’ knowledge, beliefs and attitudes towards depression and the treatment thereof have an influence on aspects, such as help-seeking behaviour or treatment adherence cf. [20, 21]. Although patients’ views may constitute potential barriers, they might also offer opportunities for managing depression. Consequently, the investigation of patient perspective seems to be essential in establishing an optimised management of depression. A qualitative research approach, in particular, allows a detailed exploration of patient perspective [22].

In contrast to the growing number of patients concerned, there are only a few qualitative studies [13, 23–29] investigating the views and experiences of patients 75 years of age or older with symptoms of depression. These studies focused on the views of, experiences with and barriers to depression treatment of minority groups or on specific aspects of the depression experience including the treatment and intervention of depression. Furthermore, these studies predominantly had broader inclusion criteria regarding age, thus patients younger than 75 years were also included. To the best of our knowledge, there has been no qualitative study investigating the views and experiences of older patients with symptoms of depression in Germany. Therefore, we conducted this study to explore the perspectives of German primary care patients 75 years of age or older regarding depression and its treatments. The aim of this study was to explore patients’ knowledge, beliefs, attitudes and experiences with depression to subsequently derive potential barriers to and opportunities for the management of depression in late life.

## Methods

This qualitative study interviewed 12 primary care patients 75 years of age or older with symptoms of depression in three German cities. The study was embedded in the multi-centre, primary care-based, cohort study AgeMooDe, which was designed to describe and compare the use of health services by primary care patients with and without symptoms of depression over the period of one year. Details of the AgeMooDe study were published elsewhere [30].

The interview participants presented here were recruited from the AgeMooDe cohort study, i.e., within a primary care setting. The criteria for inclusion in the cohort study were: age of 75 years or older and having had at least one contact with their general practitioner (GP) within the last six months - regardless of the reason for the consultation. The exclusion criteria were: severe illness (probably fatal within three months), moderate to severe dementia, insufficient ability to speak and read German, insufficient ability to consent and those patients the GP barely knew due to an ad hoc consultation. An additional inclusion criterion for the qualitative study was: only patients with a score of ≥6 on the Geriatric Depression Scale (GDS) [31] at baseline or at the 12-month follow-up in the AgeMooDe cohort study were selected for an interview. In every case, the most recent GDS score was taken. Aiming at diversity, patients were purposively selected based on their age and gender to achieve a diverse range of ages 75 years or older. Almost all participants of the AgeMooDe cohort study had agreed to be contacted for future research. Once this criterion was also fulfilled, potential participants were contacted in writing and by telephone. If the patient was willing to participate, an interview was scheduled at the patient’s home or at the study centre. All participants were informed verbally and in writing about the nature of the study, the inclusion criteria, and the interview conditions in order to obtain their written informed consent.

### Participants

Table 1 presents the characteristics of the study participants. The patients were between 77 and 91 years old (median: 81 years). According to GDS, all participating patients with a GDS score of 6 to 12 and a median GDS score of 8.5 showed symptoms of depression. During the interviews, nine patients described that they were currently suffering from depression or had suffered from it at one point in time and that they were receiving treatment. Three patients said that they have had no personal experiences with depression so far. Despite a GDS score ≥ 6 indicating underlying symptoms of depression, these patients did not identify themselves as suffering from depression.

**Table 1:** Characteristics of study participants

ID	Gender	Age	Marital status	Living situation	Education level (CASMIN)^a^	GDS Score^b^	Personal experiences with depression
Patient A	female	75–80	divorced	alone	Intermediate vocational qualification	7	Yes
Patient B	male	>80	widowed	alone	Higher tertiary education	9	No^c^
Patient C	male	>80	married	with partner	Basic vocational qualification	11	Yes
Patient D	female	>80	widowed	alone	General elementary education	6	Yes
Patient E	male	>80	married	with partner	General maturity certificate	10	No^c^
Patient F	female	>80	widowed	alone	General maturity certificate	8	No^c^
Patient G	male	>80	married	with partner	Vocational maturity certificate	7	Yes
Patient H	female	75–80	married	with partner	Higher tertiary education	10	Yes
Patient I	female	75–80	married	with partner	Basic vocational qualification	9	Yes
Patient J	female	>80	widowed	alone	Basic vocational qualification	6	Yes
Patient K	male	75–80	married	with partner	Lower tertiary education	12	Yes
Patient L	female	75–80	widowed	alone	Basic vocational qualification	7	Yes

^a^Classification according to the CASMIN Educational Classification [53]

^b^The GDS-Score was documented <1–9 months before the qualitative interview (median 2.0)

^c^Three patients said during their interview that they have had no personal experiences with depression so far. Despite a GDS score ≥ 6, they did not identify themselves as suffering from depression

### Data collection

Semi-structured interviews guided by an interview guide were conducted in February and March 2014. This type of interview allowed for a certain structure in terms of questions which were of interest but always allowed the interviewer to vary the structure of the interview guide according to the interview situation [32]. A first draft of the interview guide was literature based e.g., [27, 33–36] and developed by an experienced psychologist which was then discussed in a working group of academics and clinicians focusing on qualitative research methods. The interview guide addressed the following topics: the patients’ general knowledge of and beliefs about depression, their knowledge of and beliefs about the aetiology of depression and treatment possibilities, as well as their attitudes on how to deal with depression and people with depression. The guide also included questions about the participants’ perceptions of what other people thought about people with depression, questions about their opinions on different treatment possibilities of depression, as well as questions about potential personal experiences with depression (see Additional file 1). Four interviews were conducted in each of the following cities: Hamburg, Mannheim and Bonn. With the exception of one interview, all interviews were carried out at the patients’ homes. One interview was conducted at the Institute of General Practice/Primary Care of the University Medical Center Hamburg-Eppendorf. The interviews lasted between 31 and 93 min with a median of 47 min. All interviews were audiotaped and transcribed verbatim by trained research assistants.

### Data analysis

The interviews were analysed with MAXQDA 11 software using qualitative content analysis. This method is appropriate to systematically describe the meaning of specific qualitative material [37, 38]. First, each interview transcript was read completely to get an overall impression; consequently, interesting aspects had already been highlighted and memos had been written. Additionally, a summary containing important issues was drawn up for each interview. Thereafter, text segments were highlighted and assigned to the categories of a coding frame, consisting of main and subcategories. The topics included within the interview guide provided a framework for the inductive analysis based on the interview data. The analysis was performed predominantly by AS. To ensure a high quality analysis, the coding process was discussed regularly with HK and KH. In case of different data interpretations, consensus was sought and achieved. Data saturation, i.e. no new categories emerged in the analysis [39], occurred after the 10th interview as the interviews no. 11 and 12 only added minimal new information.

## Results

**Fig. 1:**
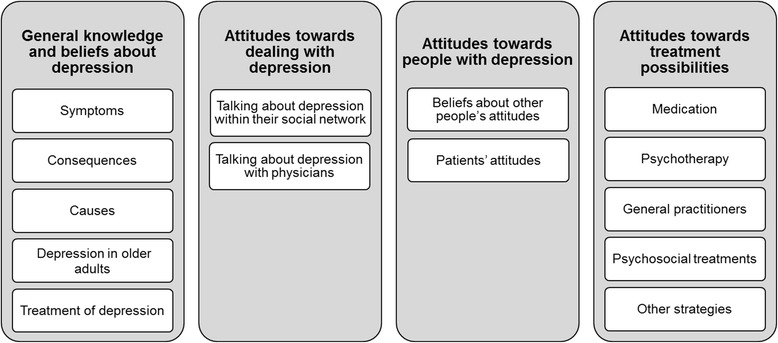
Thematic fields and their categories derived from the qualitative interviews

Figure 1 gives an overview of the 14 categories grouped into four thematic fields deriving from the analysis of the interviews. Participants’ experiences with depression and its treatment were woven into the respective categories; no additional categories were created to solely include the personal experiences of the participants because beliefs, attitudes, knowledge and experiences were always interconnected.

### Patients’ general knowledge and beliefs about depression

#### Symptoms, consequences and causes

Participants described a variety of different symptoms of depression, such as, withdrawal, listlessness, exhaustion, lacking the courage to live or sadness. According to the interviewees, depression can result in suicide, loss of family and work, social isolation, loss of self-esteem and the feeling that the affected person is no longer her- or himself. Potential causes of depression in general, mentioned by the interviewees, can be classified as “caused by life-changing events” (e.g., loss of employment or illness) or triggered by “internal causes” (e.g., predisposition or hormonal imbalances).

#### Depression in older adults

According to the interviewees, depression does not only occur at a young age but also in old age. The participants stated age -specific causes, for example, the age-related increase of illnesses and deaths amongst relatives and friends in addition to “general” causes, such as loneliness, boredom, fear of care dependency and their own death. Some participants even believed that depression occurred more often later in life because of these age-specific circumstances and fears. One interviewee, who had suffered from depression since the death of his wife, added that he had never thought about being depressed in younger years, not even during the Second World War when he had suffered a gunshot wound.

*“I’ worked all my life. I never had time to think about being depressed.” (Patient B/103).*

#### Treatment of depression

Participants varied regarding their views on the treatment of depression. A few participants believed that depression was treatable. One participant explained that his answer was based on his own experience with the successful treatment of a past depression. Several other participants believed that not every depression was equally well treatable. Thus, the treatment success depended on the severity of the depression, the patients’ readiness for treatment, whether the patient supported the treatment by being independently “active”, or the persons’ age. Answering the question: “Is depression treatable?”, an interviewee replied:

*“No, I don’t think so (…) not in my case. Not in old age anyway, maybe in my early years.” (Patient D/215–370).*

Among the participants was also the belief that the success of a treatment depended on the cause of the depression: depressions caused by life-changing events (e.g., divorce) or hormonal disorders could be treated easily, whereas depressions of unclear causes were more difficult to treat. A few other participants were unsure whether depression was treatable or whether others could help at all. One of the patients who was interviewed gave the following reason:

*“This depression, as we said, is something between heaven and earth, something not tangible.” (Patient B/408–419).*

Based on his experiences, another participant was sure that depression was not treatable. He also said that only people themselves could rid themselves from depression because it only existed in one’s mind.

### Patients’ attitudes towards dealing with depression

#### Talking about depression within their social network

It was a prominent attitude among the interviewees that they would not tell their social network- (e.g. family, friends and neighbours) when they felt depressed. At most, they would tell close relatives. Different reasons were given for this attitude: other people should not be burdened with one’s problems, participants expected negative or unhelpful responses, feelings of timidity and shame were associated with talking about such topics, or the interviewees assumed that other people were not interested in listening to their problems. A patient also said, with regard to the question whether or not she would tell her social network about being depressed:

*“Well, if it [depression] affected me, well, not right away (…) because you think you have to deal with it alone at first.” (Patient F/35–38).*

Those interviewees who reported that they would talk about depression said that the others would notice it anyway. They also stated that they were sure to find empathy, or they themselves considered it helpful to speak about it.

#### Talking about depression with physicians

Several participants affirmed that they would or did talk with a physician when they felt depressed. However, some participants said that they would not immediately address depression with a physician, they would rather first try to solve the “problem” themselves. They also said that a conversation was only possible if the physician took sufficient time and showed interest.

***Patient:*** *Yes, I would at some point [talk about depression with a physician]. Well, probably not right at the start, but I would do it.*

***Interviewer:*** *What do you think his reaction would be?*

***Patient:*** *Well, the doctors, that’s the problem, they don’t have time anymore and you would have to try and find a doctor who cares about the issue. (Patient F/41–46).*

They also expressed their concern that physicians might not pay much attention to older patients’ problems, as they regarded the treatment of older patients a waste of time. A few participants would not or did not talk with their physician when they felt depressed. On the one hand, this was due to a lack of trust and, on the other hand, due to the belief that they would not benefit from it when antidepressants had already been prescribed.

### Attitudes towards people with depression

#### Patients’ beliefs about other people’s attitudes towards people with depression

There was a widespread belief among the participants that other people had little understanding for people with depression and that depression was often not taken seriously or underrated. For example, participants believed that people with depression were still referred to as being “crazy” and that other people thought that people with depression were not ill but simply no longer wanted to work. The interviewed patients assumed the fact that depression was not visible like other disorders and that it was difficult to understand for non-involved persons were reasons for the low acceptance of depression. Particularly those who have no experience with this disorder, have little understanding.

*“People don’t think enough about it (…) what may be the cause of depression, what can be done against it. And very often it’s not even considered a problem.” (Patient K/143–144).*

#### Patients’ attitudes towards people with depression

Differently from the participants’ beliefs on what other people thought about people with depression, participants expressed an understanding for people with depression saying that they felt sorry for those people because this disorder really affected their lives. They mentioned that they would take the disorder seriously. As a reason for their understanding, some mentioned their own experiences.

*“There was no awareness of such a thing as depression a few years ago. I didn’t know anyone apart from my niece. (…) It was so terrible that she committed suicide in the end. (…) I was 72. Actually, I was annoyed because I couldn’t understand it properly. (…) But now? After more than 10 years had passed, I became depressed myself. (…) Only then could I understand her - because I had the same problem.” (Patient D/1–2).*

### Patients’ attitudes towards treatment possibilities

#### Medication

Several interviewees considered medication helpful for the treatment of depression because they themselves have had good experiences with them. However, there were also patients who believed in the effectiveness of medication without having had personal experiences. Despite a positive attitude towards medication, fears of addiction or side effects were mentioned:

*“One has to be very, very careful with medication. But without medication – it doesn’t work.” (Patient H/167–181).*

Some participants said that they could not assess the effect of medication, but interestingly enough, they were rather sceptical. For example, one interviewee thought that medication was not a causal treatment but only had a dampening effect on depression. Another interviewee said that antidepressants had a sedative effect. The negative experience of becoming addicted to pills combined with the experience that medication could not cure depression were the reasons why one participant did not believe in medication efficacy.

#### Psychotherapy

Again, patients’ opinions varied about the effectiveness of psychotherapy. A number of participants believed that psychotherapy was helpful in the treatment of depression. Similar to the medication issue, some participants based their assessment on their own or on others’ experiences.

*“I think it plays an important role in case of depression. (…) Well, in my opinion, it’s one of the key aspects.” (Patient E/129–134).*

Participants emphasised the importance of a good relationship between patient and therapist, as well as the importance of the therapist’s experience for a successful therapy. One interviewee believed that psychotherapy only made sense if the affected person was already feeling better. Another patient explained that she would like to do a second psychotherapy, but she did not know how or where to find a therapist.

At the same time, some interviewees did not consider psychotherapy helpful because of previous negative experiences or the belief that it was simply not beneficial for them, due to old age or for other reasons. Another patient was suspicious of psychotherapy. He feared that psychotherapists might manipulate people with words.

#### General practitioners

Some participants believed that a GP could only help with depression by referring the patient to a specialist or by prescribing antidepressants. In this context, patients also mentioned that they had no idea how a GP could help treat depression assuming that GPs did not know much about this disorder. The patients made this statement although several of them had mentioned having had conversations with their GPs about depression or depression associated symptoms.

*“I mean, I have a GP who, without wishing to offend him, is not a psychiatrist or something like that. Anyway, he prescribes the pills if I want them but the decision to take them is mine alone, not his. (…) I would say, (…) that he might not know that much about it anyway.”* (Patient G/62–69).

Some other participants believed or had experienced that GPs could help treat depression through conversations or counselling. On the other hand, patients criticised that conversations were restricted because of time constraints. Again, a trusting relationship and the efforts of the GP were pointed out as being important whether or not a GP was able to help treat depression.

#### Psychosocial treatments

Participants were also asked about their opinions on guided exercise programmes, animal-assisted therapy and self-help groups, which all belong to the so-called psychosocial treatment. Regarding guided exercise programmes, participants believed them to be beneficial. Distraction was emphasised as being helpful. Interestingly, one participant said that she was not going to participate in such programmes until she was in a nursing home because programmes were close by and she would not have to walk so far.

With regard to self-help groups, participants predominately believed them to be beneficial because they facilitated contact to other people suffering from depression and offered a distraction. However, some of the interviewees considered self-help groups only appropriate for people with mild depression and not an option for everyone. Interviewees also viewed animal-assisted therapy positively.

*“I have a pet. (laughs). I'd like to have a few. (...) I did have a cat… a cat and a dog. And that, I believe, is the best medicine.”* (Patient A/176–183).

#### Other strategies

Participants also mentioned other strategies on how to deal with depression. Several interviewees thought that having something to do, going out, and having contact to other people were helpful in coping with and preventing depression.

*“I know from literature that you don’t have to stay in bed and ponder all day. You can get up when you are awake. I would even say that this helps me a little.”* (Patient G/15–16).

One patient said that it was difficult for her to implement the above mentioned strategy on a regular basis because she did not have the financial resources to go swimming or out for a coffee. Praying and waiting until an episode of depression got better were experienced as being another helpful strategy. Further aspects that were considered important were the presence of a loving person, taking care of others and the feeling they got when physicians gave the impression of really understanding their psychological problems and sorrows.

## Discussion

### Main results

This qualitative study explored the views and experiences of German primary care patients 75 years of age or older with symptoms of depression. The patients in our study did not have uniform ideas about depression: we found that patients were partly well-informed about depression with positive attitudes and beliefs in treatment possibilities. However, we also found views that indicated misconceptions about depression. Another major result of our interviews was older patients’ limited willingness to talk about depression with family, friends and neighbours. As reasons for not talking about depression, participants mentioned a fear of negative reactions or the belief that others would be burdened by it. Furthermore, patients believed that other people in general had little or no sympathy for people with depression. Some patients considered the GP a contact person in case of an episode of depression and helpful in treating depression; however, our results also showed that some patients believed that GPs played an insignificant role in the treatment of depression.

### Strengths and limitations

To the best of our knowledge, this is the first qualitative study investigating patients’ views on and experiences with depression focusing on German primary care patients 75 years of age or older. However, there are limitations regarding the generalisability of our results. Firstly, all interviews were conducted with German primary care patients with symptoms of depression and living in the cities of Hamburg, Mannheim, and Bonn - each of these cities had 300,000 or more inhabitants. Patients living in rural areas might have other knowledge, beliefs, attitudes and experiences regarding depression and the treatment of depression. Secondly, we only interviewed patients who were willing to participate in an interview focusing on a topic as sensitive as depression. The views and experiences of these interviewed patients might differ from those of other primary care patients 75 years of age or older with symptoms of depression. Thirdly, we interviewed some patients who did not consider themselves as suffering from depression despite their GDS score ≥ 6. However, as the GDS had been completed between one and nine months prior to the interview, the scores might have been different at the time of the interview. Fourthly, the sample size of 12 interviews was relatively small. Although we experienced data saturation, we cannot be completely sure to have comprehensively captured the perspectives of older adults on depression and its treatment. Fifthly, by using qualitative content analysis, we were able to get a broad insight into the knowledge, beliefs, attitudes and experiences of older primary care patients with depression and the treatment thereof. To deepen the knowledge about the views and experiences of adults 75 years of age or older, we recommend further in-depth qualitative research to be able to explore the identified issues more comprehensively.

### Barriers and opportunities for the management of depression in late life

In the following text, we discuss our results with respect to potential barriers to and opportunities for the management of depression in late life identified in the patient narratives. Some of our study participants seem to be well informed about depression and its treatment and believe in the efficacy of different treatment options, such as medication or psychotherapy including psychosocial treatments. Studies have shown that positive beliefs and attitudes about treatment efficacy influence their utilization and individuals’ intention to seek help [20, 40, 41]. However, our results provide evidence that patients 75 years of age or older have also views that may have a negative impact on seeking help as well as on the acceptance of and adherence to treatments. Thus, some interviewees believed that there was no treatment for depression, that old age was a reason for reduced treatment success or that antidepressants were associated with strong side effects. Other studies also identified misconceptions about depression in older adults [13, 25, 27]. Givens et al. found that fear of addiction was the most common reason for older adults to avoid taking antidepressants [27]. However, our results indicate that past negative experiences with treatments may be an additional reason why patients do not believe in the efficacy of treatments. Consequently, this aspect also needs to be considered as a potential barrier to the management of depression. Insecurity in the search for therapists was mentioned to be a definite barrier to the use of psychotherapy.

Our results suggest that a fear of stigmatisation represents another potential barrier towards the treatment of depression. Our interviews showed that patients would rather avoid talking within their social network about depression due to shame or fear of encountering negative reactions. This fits the findings of participants’ beliefs that other people have little acceptance of people with depression, even if they themselves do accept them. An individual’s perception of other people’s stereotypes or prejudices about people suffering from depression is often referred to as a perceived (public) stigma [42–44]. Sirey et al. show that adherence to pharmacotherapy by patients with depression can be predicted by the level of perceived stigma: patients with a lower perceived stigma are more likely to show medication adherence than patients with a higher perceived stigma [21]. However, research that investigated the association of perceived public stigma with attitudes towards professional help-seeking, intention to engage or engagement in mental health treatment, or general help-seeking behaviour did not produce uniform results [42, 44–48].

Another reason why participants avoided talking about depression was the conviction that other people should not be burdened with your problems and that you had to deal with depression yourself. This belief was also present in the qualitative studies of Lawrence et al. and Switzer et al., who both identified individual responsibility of dealing with depression to be a central issue in people 65 years of age or older [28, 29]. Conforming with these studies, we also found that older adults believed in self-help by being active. However, our results indicated that the distance to exercise programs and other activities including the lack of financial resources, were potential barriers for older adults wishing to engage in such activities.

Even though several participants seemed to be open to discuss a long-lasting sadness with a physician, patients criticised that physicians often had very little time for personal conversations. Sufficient time was mentioned to be a precondition for talking about depression. Patients’ concerns that physicians might give lower priority to the problems of older adults also seemed to present a barrier. Both aspects might have influenced patients’ willingness to seek professional help when feeling depressed.

Since GPs are the main medical point of contact for older people, we investigated patients’ beliefs regarding the helpfulness of GPs. Participants mentioned various possibilities how GPs could help with depression but our results also suggested that older patients did not always consider GPs to be competent contact persons for depression assuming that GPs had limited abilities. Remarkably, this attitude was also present in participants, who apparently had talked with their GPs about psychological problems or were treated for depression.

### Implications

Our results enable us to offer suggestions for an optimised management of depression in the age group of patients 75 years or older.

Older patients may have misconceptions about depression. Age-specific information about depression in late life conveyed, for example, by information booklets, together with their GP, could be beneficial in improving older patients’ knowledge on the topic. Focus should be on information about how treatments can be used to manage depression. Particularly with respect to psychotherapy, older adults might need more support to engage in this type of treatment. Older patients with depression may have had negative treatment experiences and, therefore, may be sceptical about treatments. Information on the variety of treatments including information and discussion on how treatments have changed over the years might help patients accept a specific treatment. Furthermore, the information provided should also mention the importance of GPs as main contact persons in the treatment of depression and encourage patients to proactively communicate psychological problems with their GPs. It is also important that professionals are aware of potential fears of stigma in older patients and encourage patients to believe that depression is a recognised disorder with many treatment options. However, research is needed to investigate the effectiveness of such age-specific information on help-seeking behaviour and treatment in patients 75 years of age or older.

Patients’ perceptions that physicians are generally short of time seems to be another barrier to the management of depression. GPs in particular, who often treat comorbidities in their older patients, may agree on consultations taking the time to talk about psychological or emotional issues, contrary to consultations where physical complaints are the main reason for consultation. These “time slots” may help patients address psychological problems and allow conversations about patients’ feelings and opinions regarding a current treatment for depression.

Trying to be active is one way older primary care patients can cope with depression. They also seem to be open to psychosocial therapies. However, some patients view depression as something where others cannot help. A chance to improve depression management may be in supporting older patients with depression to help themselves. In addition to targeted information on nearby community services, collaborative care models located in the primary care setting, which also promote patients’ self-management, seem to be promising care models for older primary care patients [49–52].

## Conclusions

It is important to understand the perspectives of depression in primary care patients 75 years of age or older because patients’ beliefs and attitudes may affect treatment outcomes and help-seeking behaviour. Findings of this qualitative interview study indicate that patients’ views on and experiences with depression and its treatment methods are multifaceted: some patients seem to have positive beliefs and attitudes about depression and its treatment possibilities. However, this study also identifies views that may constitute barriers towards the management of depression in late life.

## Additional file

Additional file 1: Interview guide. Presentation of the interview guide used for the qualitative interviews. (PDF 197 kb)

## Abbreviations

GDS: Geriatric Depression Scale
GP: general practitioner

## References

[1] Gühne U, Stein J, Riedel-Heller S (2016). Depression im Alter – Herausforderungen langlebiger Gesellschaften. Depression in old age - Challenge of an ageing society. Psychiatr Prax.

[2] Volkert J, Schulz H, Härter M, Wlodarczyka O, Andreas S (2013). The prevalence of mental disorders in older people in western countries – a meta-analysis. Ageing Res Rev.

[3] Luppa M, Sikorski C, Luck T, Ehreke L, Konnopka A, Wiese B, et al. Age- and gender-specific prevalence of depression in latest-life-systematic review and meta-analysis. J Affect Disord. 2012;136:212–21. 10.1016/j.jad.2010.11.033 21194754

[4] Alexopoulos GS (2005). Depression in the elderly. Lancet.

[5] Blazer DG (2003). Depression in late life: review and commentary. J Gerontol A Biol Sci Med Sci.

[6] Fiske A, Wetherell JL, Gatz M (2009). Depression in older adults. Annu Rev Clin Psychol.

[7] Ismail Z, Fischer C, McCall WV (2013). What characterizes late-life depression?. Psychiatr Clin North Am.

[8] Pinquart M, Duberstein PR, Lyness JM (2007). Effects of psychotherapy and other behavioral interventions on clinically depressed older adults: a meta-analysis. Aging Ment Health.

[9] Wilson K, Mottram P, Sivanranthan A, Nightingale A. Antidepressant versus placebo for depressed elderly. Cochrane Database Syst Rev. 2001; 10.1002/14651858.CD000561. 10.1002/14651858.CD000561 PMC7066642 11405969

[10] Melchior H, Schulz H, Härter M. Faktencheck Gesundheit. Regionale Unterschiede in der Diagnostik und Behandlung von Depressionen 2014. https://faktencheck-gesundheit.de/fileadmin/files/user_upload/Faktencheck_Depression_Studie.pdf. Accessed 21 Dec 2016.

[11] Gum AM, Arean PA, Hunkeler E, Tang L, Katon W, Hitchcock P (2006). Depression treatment preferences in older primary care patients. Gerontologist.

[12] Crabb R, Hunsley J (2006). Utilization of mental health care services among older adults with depression. J Clin Psychol.

[13] Conner KO, Copeland VC, Grote NK, Rosen D, Albert S, McMurray ML (2010). Barriers to treatment and culturally endorsed coping strategies among depressed African-American older adults. Aging Ment Health.

[14] Gühne U, Luppa M, Stein J, Wiese B, Weyerer S, Maier W (2016). Die vergessenen Patienten – Barrieren und Chancen einer optimierten Behandlung depressiver Erkrankungen im Alter. Ergebnisse einer qualitativen Expertenbefragung. Barriers and opportunities for optimized treatment of late life depression. A qualitative analysis of expert interviews. Psychiatr Prax.

[15] Pepin R, Segal DL, Coolidge FL (2009). Intrinsic and extrinsic barriers to mental health care among community-dwelling younger and older adults. Aging Ment Health.

[16] Wuthrich VM, Frei J (2015). Barriers to treatment for older adults seeking psychological therapy. Int Psychogeriatr.

[17] Barley EA, Murray J, Walters P, Tylee A (2011). Managing depression in primary care: a meta-synthesis of qualitative and quantitative research from the UK to identify barriers and facilitators. BMC Fam Pract.

[18] Pew Research Center. Attitudes about aging: a global perspective. 2014. http://www.pewglobal.org/files/2014/01/Pew-Research-Center-Global-Aging-Report-FINAL-January-30-20141.pdf. Accessed 21 Dec 2017.

[19] Federal Statistical Office (2006). 11. Koordinierte Bevölkerungsvorausberechnung - Annahmen und Ergebnisse. Entwicklung der Bevölkerung Deutschlands bis 2050.

[20] Schomerus G, Matschinger H, Angermeyer MC (2009). Attitudes that determine willingness to seek psychiatric help for depression: a representative population survey applying the theory of planned behaviour. Psychol Med.

[21] Sirey JA, Bruce ML, Alexopoulos GS, Perlick DA, Friedman SJ, Meyers BS (2001). Stigma as a barrier to recovery: perceived stigma and patient-rated severity of illness as predictors of antidepressant drug adherence. Psychiatr Serv.

[22] Flick U (2011). An introduction to qualitative research.

[23] Black HK, White T, Hannum SM (2007). The lived experience of depression in elderly African American women. J Gerontol B Psychol Sci Soc Sci.

[24] Burroughs H, Lovell K, Morley M, Baldwin R, Burns A, Chew-Graham C (2006). Justifiable depression': how primary care professionals and patients view late-life depression? A qualitative study. Fam Pract.

[25] Conner KO, Lee B, Mayers V, Robinson D, Reynolds CF, Albert S (2010). Attitudes and beliefs about mental health among African American older adults suffering from depression. J Aging Stud.

[26] Overend K, Bosanquet K, Bailey D, Foster D, Gascoyne S, Lewis H (2015). Revealing hidden depression in older people: a qualitative study within a randomised controlled trial. BMC Fam Pract.

[27] Givens JL, Datto CJ, Ruckdeschel K, Knott K, Zubritsky C, Oslin DW (2006). Older patients' aversion to antidepressants. A qualitative study. J Gen Intern Med.

[28] Lawrence V, Banerjee S, Bhugra D, Sangha K, Turner S, Murray J (2006). Coping with depression in later life: a qualitative study of help-seeking in three ethnic groups. Psychol Med.

[29] Switzer JF, Wittink MN, Karsch BB, Barg FK (2006). "Pull yourself up by your bootstraps": a response to depression in older adults. Qual Health Res.

[30] Stein J, Pabst A, Weyerer S, Werle J, Maier W, Heilmann K (2016). The assessment of met and unmet care needs in the oldest old with and without depression using the Camberwell assessment of need for the elderly (CANE): results of the AgeMooDe study. J Affect Disord.

[31] Gauggel S, Birkner B (1999). Validität und Reliabilität einer deutschen Version der Geriatrischen Depressionsskala (GDS). Z klin Psycho.

[32] Strübing J (2013). Qualitative Sozialforschung. Eine komprimierte Einführung für Studierende.

[33] Althaus D, Stefanek J, Hasford J, Hegerl U (2002). Wissensstand und Einstellungen der Allgemeinbevölkerung zu Symptomen, Ursachen und Behandlungsmöglichkeiten depressiver Erkrankungen. Nervenarzt.

[34] Holzinger A, Beck M, Munk I, Weithaas S, Angermeyer MC. Das Stigma psychischer Krankheit aus der Sicht schizophren und depressiv Erkrankter. Stigma as perceived by schizophrenics and depressives. Psychiatr Prax. 2003;30:395–401. 10.1055/s-2003-43251 14586825

[35] Jorm AF, Christensen H, Medway J, Korten AE, Jacomb PA, Rodgers B (2000). Public belief systems about the helpfulness of interventions for depression: associations with history of depression and professional help-seeking. Soc Psychiatry Psychiatr Epidemiol.

[36] Weich S, Morgan L, King M, Nazareth I (2007). Attitudes to depression and its treatment in primary care. Psychol Med.

[37] Kuckartz U. Qualitative Inhaltsanalyse. Methoden, Praxis, Computerunterstützung. 2nd ed. Weinheim: Basel: Beltz Juventa; 2014.

[38] Schreier M (2012). Qualitative content analysis in practice.

[39] Guest G, Bunce A, Johnson L (2006). How many interviews are enough? An experiment with data saturation and variability. Field Methods.

[40] Jorm AF, Medway J, Christensen H, Korten AE, Jacomb PA, Rodgers B (2000). Public beliefs about the helpfulness of interventions for depression: effects on actions taken when experiencing anxiety and depression symptoms. Aust N Z J Psychiatry..

[41] O’Connor PJ, Martin B, Weeks CS, Ong L (2014). Factors that influence young people's mental health help-seeking behaviour: a study based on the health belief model. J Adv Nurs.

[42] Conner KO, Copeland VC, Grote NK, Koeske G, Rosen D, Reynolds CF (2010). Mental health treatment seeking among older adults with depression: the impact of stigma and race. Am J Geriatr Psychiatry.

[43] Corrigan PW, Watson AC, Barr L (2006). The self-stigma of mental illness: implications for self-esteem and self-efficacy. J Soc Clin Psychol.

[44] Vogel DL, Wade NG, Hackler AH (2007). Perceived public stigma and the willingness to seek counseling. The mediating roles of self-stigma and attitudes toward counseling. J Couns Psychol.

[45] Barney LJ, Griffiths KM, Jorm AF, Christensen H. Stigma about depression and its impact on help-seeking intentions. Aust N Z J Psychiatry. 2006;40:51–4. 10.1080/j.1440-1614.2006.01741.x 16403038

[46] Golberstein E, Eisenberg D, Gollust SE (2009). Perceived stigma and help-seeking behaviour: longitudinal evidence from the healthy minds study. Psych Serv.

[47] Pattyn E, Verhaeghe M, Sercu C, Bracke P (2014). Public stigma and self-stigma: differential association with attitudes toward formal and informal help seeking. Psych Serv..

[48] Wrigley S, Jackson H, Judd F, Komiti A (2005). Role of stigma and attitudes toward help-seeking from a general practitioner for mental health problems in a rural town. Aust N Z J Psychiatry..

[49] Chew-Graham CA, Lovell K, Roberts C, Baldwin R, Morley M, Burns A (2007). A randomised controlled trial to test the feasibility of a collaborative care model for the management of depression in older people. Br J Gen Pract.

[50] Coventry P, Lovell K, Dickens C, Bower P, Chew-Graham C, McElvenny D (2015). Integrated primary care for patients with mental and physical multimorbidity: cluster randomised controlled trial of collaborative care for patients with depression comorbid with diabetes or cardiovascular disease. BMJ.

[51] Gensichen J, Petersen JJ, Karroum T, Rauck S, Ludman E, König J (2011). Positive impact of a family practice-based depression case management on patient's self-management. Gen Hosp Psychiatry.

[52] Unützer J, Katon W, Callahan CM, Williams JW Jr., Hunkeler E, Harpole L, et al. IMPACT investigators. Improving mood-promoting access to collaborative treatment. Collaborative care management of late-life depression in the primary care setting: a randomized controlled trial. JAMA 2002;288:2836–2845. 10.1001/jama.288.22.2836 12472325

[53] Brauns H, Steinmann S (1997). Educational reform in France, West-Germany, the United Kingdom and Hungary: updating the CASMIN educational classification.

