# Supplementary material for: A qualitative study on older primary care patients’ perspectives on depression and its treatments - potential barriers to and opportunities for managing depression
Source: BMC Fam Pract. 2018 Jan 3;19:2. doi: 10.1186/s12875-017-0684-3 (PMC5751798; doi:10.1186/s12875-017-0684-3)
Supplement: Additional file 1: — Interview guide. Presentation of the interview guide used for the qualitative interviews. (PDF 197 kb) [file 12875_2017_684_MOESM1_ESM.pdf]

Interview guide

“Dear Mr/Ms [name of the interviewee],

We already know each other from a previous interview. Thank you very much for your willingness to participate in this interview as well.

Please allow me to briefly introduce myself once again. I am Mr/Ms [name of the interviewer]. I am a staff member of the research project [name of the institution] addressing issues, such as health and illnesses in older age including treatment and support options.

In this context – and this is the interview’s goal - we examine the question what you know about depressive disorders and their treatment options. You might even know someone suffering from this disorder or you yourself have experienced depressive disorders.

The knowledge about depression can differ significantly and there are totally dissimilar experiences with this disorder including treatment options. I would therefore like to give you the opportunity during this interview to tell me in detail what you know about the disorder and perhaps share your personal experience. There are no wrong answers. If I use probing questions during the interview, it is only because I would like to better understand or find out more about something you have said.

I would like to tape record the interview. Your name and the data generated will certainly be anonymised and the results will only be used for research purposes. No one will be able to trace your answers back to you.

You may take a break at any time, if you wish to do so. Just let me know. Please let me also know, when you feel like having to interrupt or stop the interview. I will gladly continue the interview at another time.

Of course, you may request that I repeat a question at any time if you did not understand or would just like me to repeat the question.

Do you have any questions for me so far?”

1. "What do you know about depression?"

Likely additional questions:

- **"What do you think it is like to suffer from depression?"**
- **"What do you think, how would you recognise symptoms of depression?"**
- **"What do you think how often depression occurs in old age?"**
- **"What do you think is the difference between depression in old age compared to depression in young adulthood?"**
- **"What do you think are the consequences of such a disorder for individuals as well as for their relatives?"**

2. "What is your opinion of people suffering from depression?"

3. "Suppose that you yourself were feeling sad or depressed over a longer period of time or were suffering from depression, how would you cope with it? What would be your opinion about yourself?"

Additional relevant questions:

- **"Would you confide in other people within your social network, such as family, friends, neighbours?"**
  - *If the answer is **NO**:*
    - **"Why not?"**
  - *If the answer is **YES**:*
    - **"What do you think their reaction would be?"**
    - **"What advice would they give to you?"**

- **"Would you tell your physician if you felt sad or depressed?"**
  - *If the answer is **NO**:*
    - **"Why not?"**
  - *If the answer is **YES**:*
    - "**What do you think his reaction would be?"**
    - **"What do you think his advice would be?"**

4. "What do you think is other people's opinion of people suffering from depression?"

Likely additional questions:

- **"What do you think are characteristics or particularities other people attribute to people suffering from depression?"**
- **"In your opinion, what do people within your social network, for example, your partner, other relatives, neighbours or friends think of people suffering from depression?"**
- **"What do you think how other people judge depression?**
- **"What do you think, how does our society judge depression?"**

5. “What do you know about the causes of depression?“
Likely additional questions:<br>– **“What do you think are likely causes or triggers of depression?“**<br>– **“What factors do you believe might play a role in a lingering depression?“**<br>– **“What factors might, in your opinion, trigger a worsening or even a relapse of depression?“**
**6. “Do you think depression is treatable?“**
*If the answer is **YES**:*<br>– **“What do you think how likely it is to be cured? Or, in other words, of 10 afflicted people, how many do you think can be cured of depression?“**<br>– **“How can you tell that a therapy is successful?“**<br>– **“What could be beneficial from your point of view?“**<br>– **“What do you think may have a negative effect on a successful therapy?**<br><br>→ *Transition to the main topic: 7. Treatment possibilities*<br>*If the answer is **NO**:*<br>– **“What do you think, why not?“**<br>– **“In what situation have you possibly, or other people you know, had a negative experience?“**<br>– **“What would you never make use of? What therapy would you refuse? Why?”**<br>– **“What do you think are the major problems in connection with a therapy?“**<br>– **“What do you think would have to be different in order for a depression therapy to be more successful?“**
**7. Treatment possibilities**
If the question has not been answered adequately: „What do you think might be beneficial?“:<br>– **“To what extent do you believe that the GP may be able to help?“**<br>– **“To what extent do you believe that other professionals, such as psychologists or psychotherapists may be able to help?”**<br>– **„How effective do you believe medication to ease the symptoms to be?”**<br>– **“How effective do you believe psychosocial therapies to be, such as self-help groups, guided exercise programmes, animal-assisted therapies?“**

8. **„Have you personally ever undergone one of the mentioned therapies?“**
If the answer is ***YES****:*<br>– **“What has proven to be particularly beneficial?**<br>– **“In your opinion, who is / was able to help the best and why?”**
9. *If the interviewee reported a personal experience with depression:*<br>– **“What are you wishing for in future therapies / over the course of the therapy?“**<br><br>*If the interviewee did not report a personal experience with depression:*<br>– **“What do you wish people suffering from depression?“**
10. **„Would you like to add something to our interview: do you think any important aspects were left out?“**
